# Surgical Decision-Making in Breast Cancer: A Retrospective Comparative Study from a Tertiary Center

**DOI:** 10.3390/jcm15103770

**Published:** 2026-05-14

**Authors:** Florin Bobirca, Dan Dumitrescu, Florentina Gherghiceanu, Anca Bobirca, Octavian Mihalache, Cristina Alexandru, Dragos Serban, Amalia Calinoiu, Raluca Boboc, Maria Sutu, Sabin Botea, Bogdan Socea, Bogdan Mastalier, Cristian Botezatu, Laura Bolovaneanu, Alberto Dulmagiu, Traian Patrascu

**Affiliations:** 1 Surgery Department, Carol Davila University of Medicine and Pharmacy, 050474 Bucharest, Romaniadragos.serban@umfcd.ro (D.S.); bogdan.socea@umfcd.ro (B.S.);; 2 Surgery Department, Dr. Ion Cantacuzino Clinical Hospital, 011437 Bucharest, Romania; 3 Marketing and Medical Technology Department, Carol Davila University of Medicine and Pharmacy, 050474 Bucharest, Romania; 4 Internal Medicine and Rheumatology Department, Dr. Ion Cantacuzino Clinical Hospital, 011437 Bucharest, Romania; 5 Internal Medicine and Rheumatology Department, Carol Davila University of Medicine and Pharmacy, 050474 Bucharest, Romania; 6 Internal Medicine Department, Prof. Dr. Agrippa Ionescu Clinical Emergency Hospital, 011356 Bucharest, Romania; 7 Department of Surgery, Sf. Pantelimon Clinical Emergency Hospital, 021659 Bucharest, Romania; 8 General Surgery Clinic, Colentina Clinical Hospital, 020125 Bucharest, Romania; 9 Department of Infectious Disease, Victor Babes Clinical Hospital for Infectious Diseases, 030303 Bucharest, Romania

**Keywords:** breast cancer, sentinel lymph node biopsy, mastectomy, general surgery

## Abstract

**Background/Objectives**: The main objective of the study was to determine the frequency of patients who underwent breast-conserving surgery (BCS) and those with modified radical mastectomy and to compare the clinical–paraclinical parameters between these groups. **Methods**: We conducted an observational, retrospective study, which included 101 patients diagnosed with breast cancer that had surgical interventions between January 2024 and April 2025. **Results**: The BCS category was represented by 36.6% cases, while 63.4% were in the mastectomy subgroup. Hemoglobin at the time of admission had an average of 13 g/dL, the difference between the two categories of patients being statistically significant. (13.7 vs. 12.7, *p* = 0.010). **Conclusions**: Although it has been a hotly debated topic in recent years, the choice of surgical technique for breast tumors still presents novelties and remains a subject of interest within surgical specialties. Selection criteria such as disease stage, histopathological subtype, and the intervention chosen by the surgeon may vary and oncological results may be comparable.

## 1. Introduction

### 1.1. Epidemiology

Breast cancer is the most common female malignancy and a major public health challenge, with over 2 million new cases and approximately 670,000 deaths annually worldwide [1]. Recent epidemiological data from Romania ranks breast cancer first among female malignancies (26.8% of all cases), confirming its dominant role in oncological morbidity and justifying the importance of continuous optimization of therapeutic strategies [2]. Recent studies state that tumor stage, molecular subtype and hormone receptors, together with demographic and lifestyle factors, influence mortality in breast cancer, while multimodal treatments (surgery, radiotherapy, chemotherapy and targeted therapies such as trastuzumab) contribute to improving the prognosis [1]. Also, advances in molecular pathogenesis and screening techniques such as digital breast tomosynthesis, magnetic resonance imaging, and artificial intelligence-assisted approaches open new perspectives towards the development of personalized therapies and precision preventive strategies [3].

### 1.2. Pathology of Breast Cancer

The pathogenesis of breast cancer is governed by a complex network of molecular and cellular processes that reflect the heterogeneity of the disease. Genetic anomalies, coupled with the activation of oncogenic pathways, lead to unregulated proliferation, enhanced cell survival, and distant metastasis.

At the same time, mitochondrial dysfunction and alterations in energy metabolism, including perturbations of oxidative phosphorylation and production of reactive oxygen species, are implicated in the evolution and aggressiveness of breast tumors [4,5]. Ultimately, these disparities in the tumor microenvironment and the ability of cells to evade immune responses determine both clinical variability between subtypes and different therapeutic responses [3].

### 1.3. Histopathology

Histopathological diagnosis is the foundation of breast neoplasm classification, allowing the identification and differentiation of subtypes with prognostic and therapeutic relevance [6]. Breast cancer is an epithelial adenocarcinoma that can be initially classified, from a histopathological point of view, into neoplastic lesions in situ and invasive carcinoma, depending on the presence or absence of breast stromal invasion.

Various in situ lesions comprise: Ductal carcinoma in situ (DCIS), which is defined by the atypical proliferation of epithelial cells within the ducts, without the invasion of the basement membrane. Lobular carcinoma in situ (LCIS), which is defined by atypical proliferation confined to the terminal lobules. The following subtypes are predominant within the invasive carcinoma category: invasive ductal carcinoma of nonspecific type (NST), constituting the predominant category of invasive tumors and invasive lobular carcinoma, which is identified by malignant discohesive cells.

In addition to these main entities, there is a large number of special histological types, for example: tubular, mucinous, papillary, or metaplastic carcinoma, each with distinct morphological features and specific prognostic implications [7].

- Immunohistochemistry (IHC) is a technique for the characterization of invasive breast cancer variants. The method relies on identifying the expression of specific proteins on tumor cells through labeled antibodies, yielding prognostic and predictive information [8] (ex: estrogen receptor (ER), progesterone receptor (PR), human epidermal growth factor receptor 2 (HER2), and Ki-67 index).

ER and PR are receptors involved in the regulation of hormone-dependent cell proliferation, and their presence indicates a favorable response to endocrine therapy, therefore, a better prognosis. In contrast, HER2 expression is associated with a more aggressive behavior but offers the option of targeted therapy. An increased Ki-67 index reflects accelerated tumor proliferation and is associated with a poorer prognosis [9,10].

The differential expression of receptors is the basis for the molecular classification of breast cancer into four main subtypes:

1. Luminal A breast cancer is characterized by ER/PR positive, HER2 negative, and low proliferation, being associated with a favorable prognosis.
2. Luminal B breast cancer maintains positive ER/PR hormonal expression, but exhibits increased proliferation and/or HER2 positivity, having a more aggressive biological behavior.
3. HER2-enriched breast cancer is defined by overexpression of HER2 in the absence of hormone receptors.
4. Triple-negative breast cancer is characterized by the lack of ER, PR, and HER2 expression, being associated with a more reserved clinical evolution [10].

### 1.4. Evolution of Breast Cancer Surgery

The evolution of breast cancer surgical treatment illustrates the progressive transition from extensive interventions to de-escalation strategies adapted to tumor biology. At the end of the 19th century, the Halsted radical mastectomy represented the therapeutic standard, involving en bloc excision of the mammary gland, pectoral muscles, and axillary lymph nodes, according to the theory of progressive locoregional dissemination. Although this approach ensured rigorous local control in an era without adjuvant therapies, it was associated with considerable morbidity, including lymphedema and functional deficit of the upper limb [11].

Later, the modified Madden radical mastectomy preserved the pectoral muscles while maintaining axillary dissection, thus reducing morbidity without compromising oncological radicality, becoming for many decades the standard procedure in operable disease, a fact also reflected in current clinical practice, where it remains an option used in selected cases [12].

**Figure 1:**
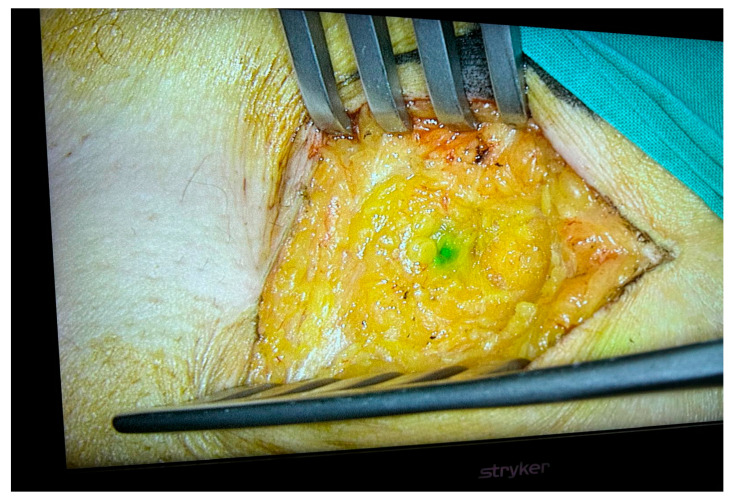
Sentinel lymph node identification with ICG.

**Figure 2:**
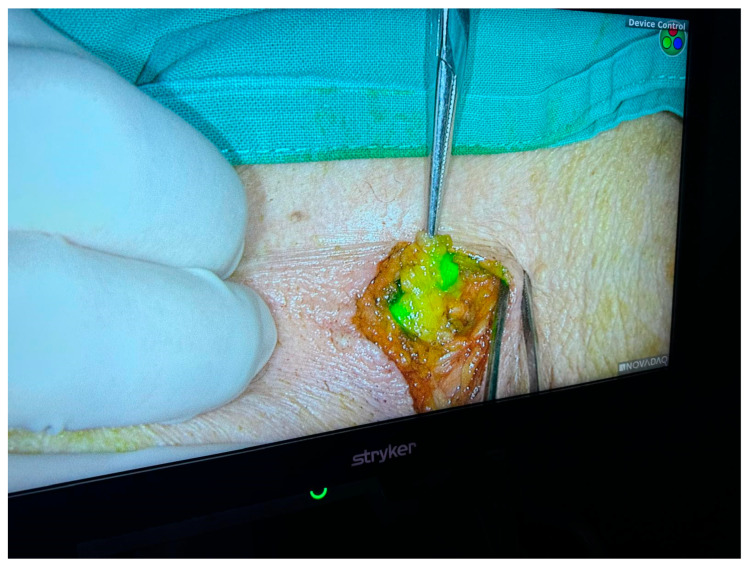
Axillary sentinel lymph node.

Advances in the understanding of tumor biology and the results of clinical trials led to the introduction of the sentinel lymph node biopsy technique, which allows for the assessment of axillary status by analyzing the first tumor-draining node. (Figure 1 and Figure 2) This method significantly reduced the need for complete axillary dissection in patients without clinical nodal involvement, marking a major paradigm shift: from systematic radical surgery to a conservative, individualized, and morbidity-reducing approach without compromising oncological safety [12,13,14].

#### 1.4.1. Mastectomy–Breast-Conserving Surgery Indications

At present, breast-conserving surgery (BCS) refers to multiple surgical procedures including lumpectomy, quadrantectomy, segmental mastectomy, or partial mastectomy. The definition of breast-conserving surgery includes removing cancer or any present abnormal tissue from the breast and a tissue excision with a safe margin while preserving the rest of the breast [15,16].

The quadrantectomy procedure involves an excision of the quadrant in which the tumor is decompressed, including a 2 to 3 cm margin, pectoralis fascia, and the overlying skin. Lumpectomy consists of tissue excision with a 1 cm safe margin [16,17].

Indications for breast-conservative surgery include:

- Early stages of breast cancer;
- Refusal of mastectomy due to near-disappearance of the tumor after neoadjuvant chemotherapy [18]

**Figure 3:**
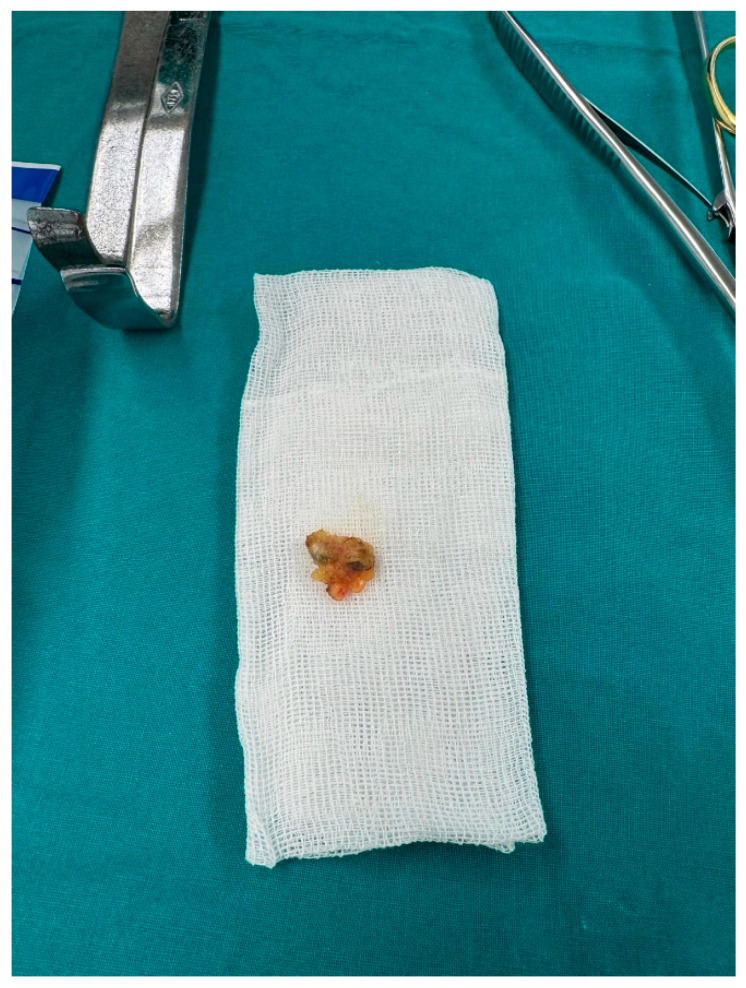
Ex vivo sentinel lymph node.

Lumpectomy is commonly recommended for DCIS/Tis and T1 and T2 tumors as long as it does not imply other contraindications to adjuvant radiation. (Figure 3).

Most recent studies have addressed the role of breast-conserving surgery referring to neoadjuvant chemotherapy for lesions that are greater than 5 cm. Neoadjuvant chemotherapy is showing comparable rates of overall survival and disease-free survival when compared to adjuvant breast cancer therapy. Patients with large tumors could be converted for breast conservation therapy [19].

The surgical procedure that is designed to remove all breast tissue is mastectomy. Simple mastectomy refers to the excision of all breast tissue, the nipple–areolar complex and skin.

Simple mastectomy is generally indicated for breast cancer patients where conservation therapy is contraindicated or it is the patient’s preference. Indications for simple mastectomy include:

- Multifocal ductal carcinoma in situ.
- Invasive breast cancer.
- Prior radiation history to the breast or chest wall.
- Prophylactic breast removal.
- Involvement of the chest wall or skin.
- A large tumor relative to breast size, making breast-conserving surgery unfeasible.
- Persistent positive margins despite multiple surgical excisions.
- Choosing to forego radiation therapy.
- Palliative treatment for locally advanced breast cancer.
- In the context of treating gender dysphoria [20].

A modified radical mastectomy implies breast tissue removal with the dissection of axillary lymph nodes for the treatment of specific types of breast cancer with lymph node involvement.

Indications for modified radical mastectomy for breast cancer include:

- Anaplastic (high-grade) invasive carcinoma.
- Clinically positive lymph nodes.
- Presence of more than three positive sentinel lymph nodes.
- Regional recurrence in the axillary lymph nodes.
- Contraindications to radiation.
- Failure to identify the sentinel lymph node [20].

#### 1.4.2. Postoperative Complications

Historically, mastectomy was the primary surgical treatment of choice [21]. Although mastectomy is still an option, breast-conserving therapy (BCS plus radiotherapy) is now recognized as an effective option, achieving comparable long-term survival in carefully chosen patients [22]. Multiple randomized trials and long-term cohort studies have confirmed equivalent overall and disease-free survival outcomes for BCS and mastectomy in early-stage disease [23].

Regarding complications of these surgical procedures, commonly reported postoperative complications following breast cancer surgery for Ipsilateral Breast Tumor Recurrence (both repeat BCS and mastectomy) are represented by wound infections, hematoma, seroma, fat necrosis, skin flap, and breast pain [24]. Severe complications are observed in 5.9% of patients undergoing breast surgery, and according to the GlobalSurg Collaborative, 36.1% of patients experience at least one postoperative complication. Most studies suggest that postoperative complications appear to be more frequent after mastectomy than after BCS [25].

Postoperative complications associated with breast surgery without reconstruction include infection, bleeding, and seroma formation. These rates are low, ranging from 2% to 50%. They are more common when the procedure is performed in conjunction with axillary surgery and immediate breast reconstruction. Complications depend on multiple factors and risks: preoperative planning, the use of prophylactic treatment, mitigation of patient-related risk factors, and surgical technique. Sentinel lymph node biopsy (SLNB) and axillary lymph node dissection (ALND) represent the two primary surgical approaches for axillary staging and management [26]. Postoperative complications associated with these procedures include seroma formation, infection, lymphedema, nerve injury, and morbidity affecting the shoulder and arm [27].

The main objective was to evaluate the frequency of patients who underwent breast-conserving surgery (BCS) and those with modified radical mastectomy and to determine the characteristics of these patients, thus providing a real-word overview, helping clinicians in making the decision regarding the type of surgery.

## 2. Materials and Methods

We conducted an observational, retrospective study, reported in accordance with the STROBE guidelines, which included patients diagnosed with breast cancer and hospitalized for breast surgery in the Surgery Clinic of the Dr. I. Cantacuzino Clinical Hospital in Bucharest, between January 2024 and April 2025.

Inclusion criteria were: age over 18 years, histopathological diagnosis of a subtype of breast cancer, and being hospitalized for breast surgery, either conserving surgery or radical surgery (modified radical mastectomy).

Exclusion criteria were: patients with distant metastases (M1), age under 18 years, and patients with a performance status index that contraindicates general anesthesia.

Data regarding demographic information, as well as specific diagnosis characteristics (histopathological examinations, tumor stage, and laboratory and paraclinical investigations) were collected from patients’ charts. Missing data was reported for the following parameters: histological diagnosis (N = 72), laterality (N = 87), imaging examen (N = 92), histological stage, and TNM grade.

Depending on the surgery, the cohort was divided into two subgroups: BCS group and radical surgery group, and comparison analyses were done.

Statistical analysis was performed using IBM SPSS Statistics 25 and Microsoft Office Excel/Word 2025. Quantitative variables were tested for distribution using the Shapiro–Wilk test and were expressed as means with standard deviations (or medians with interpercentile intervals, depending on the distribution). Quantitative variables with normal distribution were compared between study groups using the Student test (based on the equality of variances observed through the Levene test) and quantitative variables with non-parametric distribution were compared between study groups using the Mann–Whitney U test.

Meanwhile categorical variables were expressed as absolute values or percentages and comparison between the two subgroups was done using Fisher’s Exact Test.

The statistical significance threshold used in this study was α = 0.05.

It should be noted that all patients gave their consent for data processing after signing the informed consent and the approval of the ethics committee was obtained for the publication of the study. The study was conducted in accordance with the Declaration of Helsinki, and the protocol was approved by the Ethics Committee of 5173 on 20 March 2026.

## 3. Results

A total of 101 patients were included; the general characteristics of the patients were analyzed, and the results are presented in Table 1. The proportion of patients with BCS was 36.6% (N = 37) while with radical surgery it was 63.4% (N = 64). The median age was higher in the conservatively treated group compared to the radical one (65 vs. 62.5 years); statistically insignificant difference (*p* = 0.649).

The predominant environment of origin was the urban one (60.4% of cases), an aspect emphasized by both categories of patients (67.6% vs. 56.3%). Regarding the gender of the patients, the majority of the cohort were female with a single male participant described in the conservatively treated group (100 cases, 99%, *p* = 0.366).

Regarding histopathological diagnosis before surgery, in both subgroups, the most common was invasive breast carcinoma, representing the majority of cases with a total of 51.4% of cases, with the subcategories of treatment (52.9% vs. 50.9%) (conservative vs. radical), followed by invasive ductal carcinoma with 27.8% of cases (35.3% vs. 25.5%), invasive lobular carcinoma with 15.3% of cases (5.9% vs. 18.2%), and invasive mucinous carcinoma with 5.6% of cases (5.9% vs. 5.5%). The laterality of the tumors revealed that distribution was almost in equal proportions (49.4% vs. 50.6%), with a slight predominance on the left side in those treated conservatively (54.5% vs. 45.5%).

Table 2 presents the medical history and comorbidities of the examined patients, with no significant differences between the two subgroups. Previous breast surgery was present in 14 cases, seven in each subgroup (*p* = 0.370). The most common cardiovascular diseases were represented by hypertension in 56.4% of cases (59.5% vs. 54.7%), followed by atrial fibrillation in 5.9% of cases, stroke in 5% of cases, and ischemic cardiomyopathy in 4% of cases. Among the metabolic disorders, type II diabetes mellitus was noted, present in 11.9% of cases (8.1% vs. 14.1%, *p* = 0.528).

Table 3 highlights both the particularities related to laboratory tests and imaging exams. Hemoglobin at the time of admission had an average of 13 g/dL (IQR 12.3–14.1), the subgroup who underwent radical surgery having a significant lower value (13.7 vs. 12.7, *p* = 0.010). The level of tumor marker specific to breast tumors, CA 15-3, was lower in patients treated conservatively than in those with radical treatment (15.3 vs. 20.8, *p* = 0.085).

Breast ultrasound was the most frequently used imaging investigation in this study, with 69.6% of cases being evaluated through this procedure, with a significantly higher rate among patients with conservative surgery (93.9% vs. 55.9%, *p* < 0.001), while, 77.4% of patients had a Computer Tomography (CT) examination, with an increased frequency among patients with radical surgery (51.5% vs. 91.7%, *p* < 0.001).

Mammography was done on 63.0% participants of the entire group (58 cases), while breast MRI was used only in 22.8% participants (21 cases).

Tumor staging is presented in Table 4. Histological grading highlighted the fact that in the conservatively treated group there was an equal distribution of the three categories, each represented by 33.3%. In contrast, radically treated patients were mostly classified as G2 (53.1% of cases), followed by G3 (30.6%) and G1 (16.3%), respectively. Within the TNM classification, stage T2 was the most common (72.2% of cases), followed by T3 (16.7%), T1, and T4 (each with 5.6%).

The rate of postoperative complications (seroma, postoperative hemorrhage) was 10.1%, more common in the radically treated group compared to the conservatively treated group (14.3% vs. 2.8%, *p* = 0.088). Regarding hospitalization period, a longer period was noted in the radically treated group compared to the conservatively treated group, 7 vs. 4 days; a statistically significant difference (*p* < 0.001).

## 4. Discussion

The choice of surgical intervention between modified radical mastectomy and breast-conserving technique has been a topic of intense debate in the literature for several decades. The first criterion related to this aspect is that of age. A study from Denmark that divided the patient group into age groups highlighted the fact that the 55–64 year-old group is the most numerous (17,533 cases, 30.05%) [28], as well as a Dutch study conducted over a period of over 10 years with a group of 37,207 patients showing that the 50–69 age group accounted for 41% of cases with radical treatment and 58% of cases treated conservatively [29], a result that is confirmed in our study with a mean age of 63 years.

Depending on the histopathological subtype, a predominance of the invasive ductal carcinoma subtype (95.6%) was noted in the literature in a study published in 2021 in China [30], different from what we obtained in the present work—27.8% of cases, but without a statistically significant value (*p* = 0.601). On the other hand, another work with a group of 16,522 patients suggests a predominance also in the direction of invasive ductal carcinoma in both conservatively treated and radically treated cases (92.7% vs. 89.8%, *p* < 0.001), but with a laterality comparable to the study carried out by us (51.0% vs. 49.0% in the mastectomy group, 50.1% vs. 49.9% in conservative interventions) [31].

Cardiovascular diseases in patients with breast cancer were studied by I. Engebretsen in a study based on a group of 27,535 patients with an average age of 62 years, comparable to the present study, determining arterial hypertension in 11.61% of cases and atrial fibrillation in 4.80% of cases [32].

Preoperative levels of hemoglobin are presented in a study conducted on a group of 2123 patients who had surgical interventions for breast tumors, also divided into two subgroups with radical and conservative interventions, respectively, the result being that 99.2% of the tumors in which mastectomy was performed had hemoglobin below 12 g/dL, unlike the cases proposed for breast-conserving surgery which had a percentage of 0.7% [33].

Another aspect taken into account in this pathology was tumor grading, Marissa C van Marren and collaborators noting that in the group evaluated over 10 years in patients with breast cancer, the G2 subtype was predominant (41% vs. 40%) in both types of interventions, a result superimposable with the present work in which the same category was represented in a proportion of 33.3% vs. 53.1%, but without statistical significance (*p* = 0.442) [29].

The HER2 receptor status was carefully emphasized in the work of Christiansen P., Thus it was observed that 12% of cases treated via breast-conserving surgery were positive, as well as in 24% of those with mastectomy, a conclusion that differs from our results—37.5% vs. 29.3%. If we also consider estrogen receptor (ER), then we notice a smaller difference between the two studies, 74% vs. 83% in the Danish study compared to 100% vs. 79% in our study, the differences that are not statistically significant. (*p* = 0.687, *p* = 0.322) [28] Likewise, in a study conducted on a cohort of 5335 patients with breast cancer in which the same two categories of interventions are compared, positive ER status was observed in 44.6% of cases treated with breast-conserving surgery versus 46.5% of cases treated with mastectomy, with statistical significance (*p* < 0.0001) [34].

The present study reflects a predominance of cases of advanced disease, respectively of less favorable anatomical conditions, highlighting the fact that radical interventions prevail over BCS. (63.4% vs. 36.6%) Another argument in this regard is represented by more detailed imaging investigations, such as CT, being more often used in the case of patients who were selected for radical intervention compared to the other subgroup. (91.7% vs. 51.5%, *p* < 0.001). These results are consistent with the specialized literature where mastectomy is indicated for multifocal tumors or bulky tumors relative in relation to the remaining breast volume.

The shorter hospitalization period associated with the BCS group (4 vs. 7 days, *p* < 0.001) translates into faster postoperative recovery. The relevance of this result can be interpreted as faster social reintegration of patients and lower use of medical resources, as well as reduced surgical trauma.

Likewise, the higher rate of postoperative complications in the radically treated group (14.3% vs. 2.8%) correlates with higher morbidity, associated with a more aggressive dissection in the lymph node region of this lymph basin. This aspect confirms the current trend of de-escalation of axillary surgery, especially when the SLNB technique begins to have more indications, being associated with a lower rate of complications, without altering the results from an oncological point of view.

The difference in hemoglobin levels between the two subgroups is statistically significant (13.7 vs. 12.7, *p* = 0.010), indicating that patients with tumors operated through BCS had a lower systemic impact, consistent with the literature that associates disease at a more advanced stage with a degree of anemia.

The main contribution of the study is not so much in the depth of the observed associations, which are largely reflected in the specialized literature, but rather in the fact that it offers a contemporary, real-life description of how clinical, imaging and laboratory variables are managed regarding the surgical decision between BCS and mastectomy in a Romanian tertiary center. Most studies with large cohorts come from North America, Western Europe, and East Asia, and comparisons with Eastern Europe are rare. The results that state that the local distribution is moving towards mastectomy, compared to countries with a more elaborate screening program, and also reflect a more advanced stage of the disease at the time of presentation.

Although the results present relevant information for surgical practice, their analysis will be based on the methodological limitations of the study. The primary limitation is due to the retrospective, real-world nature of our study. The data was collected from hospital records over an extended period during which documentation practices, diagnostic protocols, and access to immunohistochemical testing were not uniform. In particular, some patients were diagnosed or initially treated in other institutions, and complete pathological or staging information was not always transferred to our center. Additionally, earlier cases predate the routine standardization of certain biomarkers, contributing to the observed gaps. Secondary, the small size of the group may lead to an underestimation of statistically significant results and a decrease in the robustness of the final results. Furthermore, As the study is based on the experience of a single surgery clinic, a potential institutional bias can be inferred, reflecting local particularities of the experience of the surgical teams or access to adjuvant therapies. Another limitation that we emphasize with is the lack of follow-up—without the detection of distant relapses and survival analysis, the work is limited to perioperative and immediate postoperative results. Despite these limitations, we believe it is important to retain and analyze these data. Real-world datasets inherently reflect such imperfections, yet they provide valuable insight into clinical decision-making outside controlled trial settings. Excluding cases with missing data would further reduce the sample size and may introduce selection bias, potentially distorting the representation of actual practice. One important aspect for us is to describe patterns of surgical decision-making, and the available data remain informative for this purpose, so we can further improve our decision regarding surgery type and provide a better outcome for our patients. Furthermore, future studies based on larger, more comprehensive datasets are essential for better understanding decision-making in breast cancer surgery. In our opinion, the next step in our research agenda is identifying independent predictors, which are crucial not only for optimizing individualized treatment strategies, but also for reducing variability in care and aligning clinical practice with evidence-based guidelines.

## 5. Conclusions

This study shows a preference for radical surgery over conservative procedures in patients with breast cancer, with the former being chosen in more than half of the cohort. Furthermore, conservative techniques were associated with a lower complication rate, as well as a shorter duration of hospitalization, being an additional argument for this option in selected cases. These observations reiterate that conservative treatment could be a safer option due to the lower associated morbidity, with the mention that it respects oncological criteria.

A very important aspect in decision-making regarding breast cancer surgery is the multidisciplinary approach, as some cases are complex, involving patients with comorbidities and other medical conditions that must be carefully weighed to ensure appropriate and individualized management. However, the small sizeof the cohort, the retrospective type of the study, and the lack of patient follow-up empathizing the need to further investigate this topic on a larger scale so we can identify specific factors that can guide the choice of surgical approach between conservative or radical treatment in breast cancer.

## Figures and Tables

**Table 1:** General characteristics of the cohort. * Mann-Whitney U Test, ** Fisher’s Exact Test, NST=.

Parameter	Total	Conservative	Radical	*p*
**N, %**	101 (100%)	37 (36.6%)	64 (63.4%)	-
**Age (median (IQR))**	63 (54.5–69)	65 (54.5–68)	62.5 (54.25–70)	0.649 *
**Environment (urban) (No., %)**	61 (60.4)%	25 (67.6%)	36 (56.3%)	0.297 **
**Sex (female) (No., %)**	100 (99%)	36 (97.3%)	64 (100%)	0.366 **
**Length of hospitalization (median (IQR))**	6 (4–9)	4 (2–6)	7 (5–10)	<0.001 *
**Histological Diagnosis (No., %) (N = 72)**				
**Invasive ductal carcinoma**	20 (27.8%)	6 (35.3%)	14 (25.5%)	0.648 **
**Invasive lobular carcinoma**	11 (15.3%)	1 (5.9%)	10 (18.2%)	
**Invasive carcinoma NST**	37 (51.4%)	9 (52.9%)	28 (50.9%)	
**Invasive mucinous carcinoma**	4 (5.6%)	1 (5.9%)	3 (5.5%)	
**Laterality (No., %) (N = 87)**				
**Right**	43 (49.4%)	15 (45.5%)	28 (51.9%)	0.660 **
**Left**	44 (50.6%)	18 (54.5%)	26 (48.1%)	

**Table 2:** Medical history of patients. ** Fisher’s Exact Test.

Comorbidities (No., %)	Total	Conservative	Radical	*p*
**Hypertension**	57 (56.4%)	22 (59.5%)	35 (54.7%)	0.681 **
**Diabetes mellitus**	12 (11.9%	3 (8.1%)	9 (14.1%)	0.528 **
**Obesity**	19 (18.8%)	7 (18.9%)	12 (18.8%)	1.000 **
**Atrial fibrillation**	6 (5.9%)	2 (5.4%)	4 (6.3%)	1.000 **
**Stroke**	5 (5%)	3 (8.1%)	2 (3.1%)	0.353 **
**Ischemic heart disease**	4 (4%)	0 (0%)	4 (6.3%)	0.294 **
**Previous neoplasia**	15 (14.9%)	8 (21.6%)	7 (10.9%)	0.159 **
**Surgical interventions**	51 (51.5%)	18 (50%)	33 (52.4%)	0.837 **
**Breast surgery**	14 (13.9%)	7 (18.9%)	7 (10.9%)	0.370 **

**Table 3:** Laboratory and paraclinical examination characteristics of the cohort. * Mann-Whitney U Test, ** Fisher’s Exact Test, *** Student T-Test.

Laboratory Parameter	Total	Conservative	Radical	*p*
**Hemoglobin (median (IQR))**	13 (12.3–14.1)	13.7 (12.6–14.2)	12.7 (12.1–13.5)	0.010 *
**Leukocytes (median (IQR))**	7.3 (5.8–9.05)	7.58 (6–8.9)	6.73 (5.7–9.1)	0.343 *
**Platelets (mean ± SD)**	262.4 ± 68.5	267.6 ± 69.3	259.4 ± 68.5	0.565 ***
**INR (median (IQR))**	1.05 (1.01–1.09)	1.06 (1–1.09)	1.05 (1.01–1.09)	0.933 *
**CA 15-3 (median (IQR))**	18.4 (13.3–27.5)	15.3 (11.1–29.1)	20.8 (16–27.2)	0.085 *
**CEA (mean ± SD)**	3.24 ± 1.85	-	3.24 ± 1.85	-
***Imaging Exam (No., %)***				
**Ultrasound (N = 92)**	64 (69.6%)	31 (93.9%)	33 (55.9%)	<0.001 **
**Mammography (N = 92)**	58 (63.0%)	23 (69.7%)	35 (59.3%)	0.373 **
**MRI (N = 92)**	21 (22.8%)	8 (24.2%)	13 (22%)	0.802 **
**CT (N = 93)**	72 (77.4%)	17 (51.5%)	55 (91.7%)	<0.001 **
**Right breast BIRADS (median (IQR))**	4.5 (2.75–5)	4 (3–5)	5 (1–5)	0.552 *
**Left breast BIRADS (median (IQR))**	4 (1.5–5)	4 (1.5–4.5)	5 (1.25–5)	0.095 *

**Table 4:** Tumor staging, TNM=, ER+ = estrogen receptor, PGR+, HER2+. ** Fisher’s Exact Test.

Histologic Grade (N = 58)	Total	Conservative	Radical	*p*
**G1**	11 (19%)	3 (33.3%)	8 (16.3%)	0.442 **
**G2**	29 (50%)	3 (33.3%)	26 (53.1%)	
**G3**	18 (31%)	3 (33.3%)	15 (30.6%)	
**TNM Stage (N = 18)**				
**T1**	1 (5.6%)	1 (20%)	0 (0%)	0.299 **
**T2**	13 (72.2%)	4 (80%)	9 (69.2%)	
**T3**	3 (16.7%)	0 (0%)	3 (23.1%)	
**T4**	1 (5.6%)	0 (0%)	1 (7.7%)	
**N0**	13 (72.2%)	4 (80%)	9 (69.2%)	0.299 **
**N1**	3 (16.7%)	0 (0%)	3 (23.1%)	
**N2**	1 (5.6%)	1 (20%)	0 (0%)	
**N3**	1 (5.6%)	0 (0%)	1 (7.7%)	
**TNM stage − M0 (N = 18)**	18 (100%)	5 (100%)	13 (100%)	-
**ER + (N = 48)**	40 (83.3%)	9 (100%)	31 (79.5%)	0.322 **
**PGR + (N = 45)**	33 (73.3%)	7 (87.5%)	26 (70.3%)	0.419 **
**HER2 + (N = 49)**	15 (30.6%)	3 (37.5%)	12 (29.3%)	0.687 **

## Data Availability

The data presented in this study is available in the article.

## Abbreviations

ALND	axillary lymph node dissection
BCS	breast-conserving surgery
DCIS	ductal carcinoma in situ
ER	estrogen receptor
HER2	human epidermal growth factor 2
IHC	Immunohistochemistry
LCIS	lobular carcinoma in situ
NST	invasive ductal carcinoma of nonspecific type
PR	progesterone receptor
SLNB	sentinel lymph node biopsy
